# Book Reviews

**Published:** 1898-07

**Authors:** 


### BOOK REVIEWS.

YELLOW FEVER. Clinical Notes by Just Tonatre, M.D. (Paris), Former Physician-in-Chief of the French Society Hospital, New Orleans; Member of Board of Experts, Louisiana State Board of Health. Pp. 220. Price $2.50. Published by the *New Orleans Medical and Surgical Journal*, New Orleans, Louisiana.

This is the sort of book that the practitioner needs. It is the message of a physician to physicians, embracing the lessons derived from the author's contact and experience with yellow fever in nine epidemics, during a residence of thirty-three years in New Orleans. The following expression of a great truth is too much lost sight of in this day of wild chasing for germs: "It is at the bedside that we learn the course of the disease, the value or importance of a symptom, and the propriety or urgency of such and such medication. Hence I believe that while it is necessary to keep an eye on the microbe, the enemy, both eyes must be kept wide open on the patient, the victim. * * * Bacteriology and Practice must march hand in hand and assist each other; for, if the culture tube and the microscope increase our knowledge and make it more precise, it is Medicine which must apply the new-born truths to the cure of the patient and the clinician must remain the high priest of Medicine."

After some *general observations* the author takes up *symptomatology*, discusses each symptom, its relative frequency and importance. He lays great stress upon the value of *Faget's law* as an "indispensable diagnostic sign" during the first or second day. (This law is the coincident *fall of pulse with rise of temperature.*) An original and valuable feature of the book is a reproduction of forty-six clinical charts with clinical histories of corresponding patients. The writer makes the important observation that all the great epidemics in New Orleans have begun in May, June, or July, and all epidemics beginning in August or September have been mild and lacked virulence.

As to treatment the author does not believe in much medication. He urges: (1) absolute rest of the patient; (2) thorough aeration of the room; (3) strict personal cleanliness; (4) cold sponging for the fever, and other indications for treatment are fully discussed. The author believes that Sanarelli has discovered the germ of yellow fever and expects much from the promised curative serum.

The appearance of this book is quite timely. Every physician in yellow fever districts should have it and study it. It must remain for many years an authoritative contribution to the literature of this disease.

---

ATLAS OF LEGAL MEDICINE. By Dr. E. Von Hofmann, Professor of Legal Medicine and Director of the Medico-Legal Institute at Vienna. Edited and translated by Frederick Peterson, M.D., of New York, and A. O. J. Kelly, M.D., of Philadelphia. W. B. Saunders, Publisher, 925 Walnut St., Philadelphia. [Price $3.50 net.]

"There is perhaps no field of science in which the value of illustrations is greater than in forensic medicine. The problems which confront the coroner, the port-mortem examiner, and the courts of law must be solved by the presentation of indisputable facts. * * A volume such as this, made up chiefly of photographs and original drawings of various lesions and pathological conditions, taken directly from actual cases, supplies to every physician and student an enormous array of medico-legal data, such as would take one many years to acquire alone. This volume is a veritable treasure-house of information gained from the rich material of one of the greatest institutes of legal medicine in the world." (Translators' Preface.)

The volume contains 56 plates in colors and 193 half-tone illustrations in black. We cannot here even give a list of the subjects and conditions pictured. Among them are: Varieties of hymen, vagina and uterus after pregnancy and abortion, lungs of newly-born infants, fractures of skull, suicides by cutting throat and by strangulation, varieties of wounds, alterations of tissues due to irritant poisons, gunshot wounds of skull and brain, contusions of the brain, extra-dural hematoma, etc. The work will be exceedingly useful to the physician and it will be found a convenient supplement to some of the standard text-books on medical jurisprudence. These hand-atlases now being issued by Mr. Saunders will be of great use to the reader in helping him obtain correct pictures of various abnormal conditions.

---

DISEASES OF THE SKIN, THEIR CONSTITUTIONAL NATURE AND CURE. By J. Compton Burnett, M.D., Author of "Ringworm, Its Constitutional Nature and Cure." Third Edition, revised and enlarged. Philadelphia. $1.00. Bœricke & Tafel, 1898.

The author of this book is a homeopath with peculiar views. His home country, England, does not recognize his school.

The book is full of rubbish and is unworthy of serious consideration, save in so far as is necessary to give warning against its pernicious teachings. M. B. H.

---

LIPPINCOTT'S MAGAZINE FOR JUNE, 1897.

The complete novel in the June issue of *Lippincott's*, "As Any Gentleman Might," is a rattling tale of adventure by William T. Nichols. The hero is an American, but the action is mainly in England, and the time is the early part of the present century.

The other stories, "To Him that Hath," by Annie Nathan Meyer, and "From the Grand Stand," by Jean Wright, are very brief. The former shows how subscriptions may be won for charitable work.

"A Feathery Début," by Lalage D. Morgan, is a charming account of a family of thrushes, whose domicile was in the writer's garden. Natural history is further represented by "A Year of Butterflies," by Frank H. Sweet.

Fanny Bullock Workman describes "Spanish Plains and Sierras"; R. G. Robinson writes of "A Yankee Farmer in Florida"; and John Murdoch has some words on "Fireplaces of Snow."

"College Athletics" are vindicated by Albert Tyler, one of the American victors in the Olympian games at Athens in 1896. Francis M. Butler writes of "Teacup Times," and Edward S. Van Zile resurrects "New York's First Poet," namely, Jacob Steindam, whose works appeared in 1659 and 1661.

The poetry of the number is by Julien Gordon, Carrie Blake Morgan and Grace F. Pennypacker.

---

"The Ship's Doctor."

Intense interest to-day centers about our gallant navy, and the recent daring exploits of our sailor heroes add new luster to the brave record of the past. Americans are proud to inscribe new names standing for heroic deeds—the names of Dewey, Hobson and Powell.

Whatever tells of warships and the gallant deeds of brave sailors is eagerly perused by the American people. Our navy is the popular theme for story and picture. The brave exploits of our sailors are the absorbing topics in newspaper, review and magazine, and everywhere are seen the pictures of great battleships, graceful cruisers, of sea battles and sailor heroes.

But, numerous as are the current chronicles of sea warfare, vivid as are many of the portraitures of battle, danger and death, there has been one void in the record of heroic deeds. Deep down in the bowels of the ship there is hidden in times of battle a phase of sea life of which the world knows nothing, which has not been written of, and which artists have rarely seen or imagined. Few, indeed, are the phases of human life which have not been dissected by the literary anatomist, or fixed in vivid horror upon the canvas of the artist; and the beautiful brochure entitled "The Ship's Doctor," which is being issued to physicians by The Arlington

Chemical Co., of Yonkers, N. Y., is of unique interest. Nor is this interest due solely to the novelty of the subject, for, independently of this, the booklet is notable as marking the highest point yet reached in certain features of artistic bookmaking. The deadly battle horrors of the surgeon's merciful vocation are full of dramatic opportunities for the artist; but only an artist of power can make such gruesome scenes impressive instead of merely horrible. Mr. W. Granville Smith is such an artist, and he has made for "The Ship's Doctor" a series of battle pictures which touch the highest mark of the illustrator's art. A great naval battle is depicted with thrilling realism, and the grim realities of war are uncoverrd by portrayals of the cockpit during an action and of episodes of the surgeon's battle duties. Seldom is realism and local color, the very feeling of a scene, better rendered than in these strong drawings, and the force of the artist's work is preserved by the remarkable character of the mechanical reproduction. A marvelous advance in illustrative art has followed, and the powerful illustrations of "The Ship's Doctor" are among the most perfect examples of a beautiful new art.

The beauty of this booklet, its professional interest, and its timeliness, are certain to make a lively call for it, and physicians who have not received a copy should at once send for it, as the edition is limited and will be issued in the order as requests are received. The more important pictures are admirable subjects for framing, and if there are received a number of requests sufficient to warrant the great expense, a series of plates in large size, with liberal margins suitable for framing, will be made and supplied free to physicians. Physicians who would like to have them for framing should make their requests to The Arlington Chemical Co., of Yonkers, N. Y., makers of Liquid Peptonoids, without loss of time.

---

A TRUE SAYING.—Dr. Sinclair, of Manchester, is responsible for this *bon mot:* "Modern medical students learn surgery, which but few practice; nearly all practice obstetrics, which few learn."—*Ex.*

